# Sand fly fauna (Diptera: Psychodidae: Phlebotominae) in an area of leishmaniasis transmission in the municipality of Rio Branco, state of Acre, Brazil

**DOI:** 10.1186/1756-3305-7-360

**Published:** 2014-08-07

**Authors:** Thais Araujo-Pereira, Andressa A Fuzari, José Dilermado Andrade Filho, Daniela Pita-Pereira, Constança Britto, Reginaldo P Brazil

**Affiliations:** Laboratório de Biologia Molecular e Doenças Endêmicas, Instituto Oswaldo Cruz, Rio de Janeiro, Brazil; Laboratório de Doenças Parasitárias, Instituto Oswaldo Cruz – Fiocruz, Av. Brasil 4365, Rio de Janeiro, 21040-900 Brazil; Centro de Referência Nacional e Internacional para Flebotomíneos, Centro de Pesquisa René Rachou – Fiocruz, Belo Horizonte, Brazil

**Keywords:** Sand fly, Fauna, Amazon, Acre, Brazil

## Abstract

**Background:**

Notifications concerning American Cutaneous Leishmaniasis have increased in recent years in the state of Acre, Brazil. Despite identification of distinct *Leishmania* species isolated from cutaneous lesions, there are still no records of visceral leishmaniasis in the state. However, studies on the sand fly fauna in this region are still limited.

**Findings:**

Insects were collected from April 2011 to April 2012, using HP light traps distributed in four residential areas and one recreational area in Rio Branco, Capital of the State of Acre in the Amazon region of Brazil. A total of 456 sand flies were collected, comprising 256 females and 200 males. Taxonomic identification revealed 16 Phlebotominae genera and 23 species, as follows: *Trichophoromyia auraensis, Nyssomyia whitmani, Nyssomyia antunesi, Pressatia choti, Evandromyia saulensis, Evandromyia walkeri, Evandromyia begonae, Migonemyia migonei, Pintomyia serrana, Psychodopygus paraensis, Sciopemyia sordelii, Migonemyia pusilla, Pintomyia nevesi, Brumptomyia avellari, Micropygomyia acanthopharynx, Micropygomyia micropyga, Pintomyia odax, Lutzomyia sherlocki, Pressatia calcarata, Pressatia duncanae, Bichromomyia flaviscutellata, Evandromyia bourrouli* and *Evandromyia bacula.* From this group, *Tr. auraensis* and *Ny. whitmani* were the most abundant species in both forested areas and the peridomiciliary environment.

**Conclusions:**

We find that the sand fly fauna in the urban and peri urban areas of Rio Branco is very diverse comprising 23 species, as diverse as that in areas of primary forest. Some species, such as *Nyssomyia whitmani*, *Ny. antunesi* and *Bichromomyia flaviscutellata* are known vectors of parasites responsible for cutaneous leishmaniasis, and *Trichophoromyia auraensis* is a putative vector in this Amazonian region.

## Findings

Leishmaniasis is widely distributed in Brazil, where the cutaneous clinical form of the disease has been recorded in all states. Recent data from the Ministry of Health revealed changes in the epidemiological profile of cutaneous leishmaniasis (CL) due to its undergoing territorial expansion [1]. The disease is caused by a variety of dermotropic *Leishmania* species and a great diversity of these parasites are found in the Amazon Region. Except in primary forest areas in North Brazil and the Amazon region, *Leishmania (Viannia) braziliensis* is the main widespread etiologic agent of CL in the country [2].

The Amazon region is especially interesting due to the biological diversity of potential vectors and reservoirs, which may permit the sympatric circulation of various *Leishmania* species [3, 4]. Within this context, the Amazonian region has been identified as comprising an epidemiological circuit of rural and occupational variables that are associated mainly with the destruction of forests. Notifications of CL have increased in recent years in the state of Acre, with 2,571 confirmed CL cases recorded between 2010 and 2012 [5]. Acre has also reported a large number of mucosal leishmaniasis cases: however, the cutaneous form is still more prevalent (80%) than the mucosal form (13%), and there are no records of visceral leishmaniasis in the state [5]. The *Leishmania* species isolated from cutaneous lesions of patients from the municipality of Rio Branco were recently characterized as *L. (Viannia) braziliensis*, *L.* (*V*.) *guyanensis*, *L.* (*V*.) *lainsoni* and a hybrid of *L.* (*V*.) *naiffi* and *L.* (*V*.) *lainsoni* [6, 7].

In 2008, a previous study was conducted regarding the phlebotomine fauna from the municipalities of Bujari, Xapuri and Rio Branco. From 52 identified species, *Nyssomyia antunesi, Ny. whitmani, Ny. umbratilis, Psychodopygus davisi, Ps. hirsuta hirsuta, Ps. paraensis, Ps. ayrozai, Migonemyia migonei, Bichromomyia flaviscutellata and Trichophoromyia ubiquitalis* are known to be vectors of *Leishmania*. These data suggested the existence of three transmission cycles in Acre, including the transmission of *L. (V.) guyanensis* by *Ny. umbratilis* in the south of the Amazon River [8]. However, studies on the sand fly fauna in Acre are still limited.

### Sand fly capture and identification

Captures were undertaken in the municipality of Rio Branco, from April 2011 to April 2012, with the support of the State Health authorities. Sand flies were collected in forested areas impacted by the presence of man around residences, inside the Municipal Park and in three chicken coops present in the peridomicile of residences, using five HP light traps [9] per night during fifteen nights. Shannon trap was only used in one residence from Area II, during one night from 19 h to 21 h. Sand fly specimens were individually mounted on glass slides and the species identification followed the classification proposed by Galati [10] and species abbreviations by Marcondes [11].

### Study area

**Figure 1:**
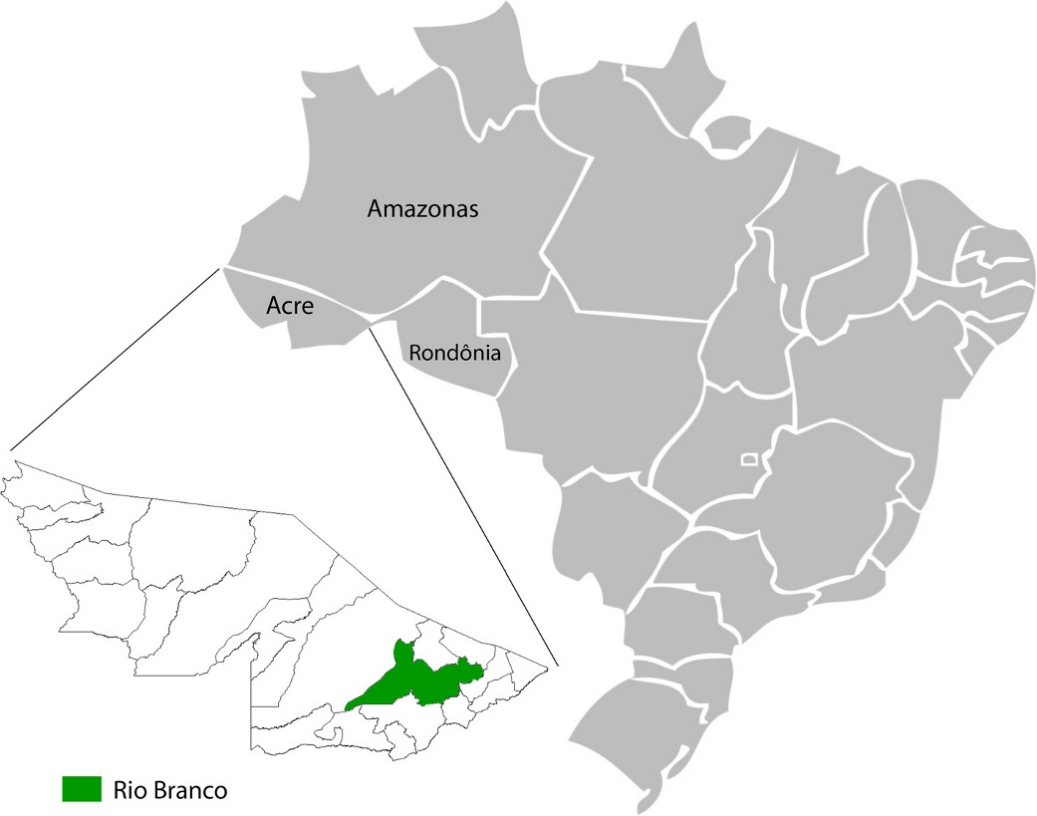
**Map of the study area.** Political map of Brazil illustrating and highlighting the state of Acre and the municipality of Rio Branco, the area of study.

The state of Acre occupies an area of 152,581 km^2^ in the north region of Brazil at the extreme west (09° 00' 00" S, 70° 00' 00" W), bordering the states of Amazonas and Rondônia, and the countries of Peru and Bolivia. Rio Branco is the capital of Acre, located in the Acre river valley (Figure 1). It is the most populated municipality in the state, with 776,463 inhabitants – almost half of the state population [12]. The city of Rio Branco has the lowest average annual temperature among the northern capitals. The climate is equatorial, with temperatures between 25°C and 38°C during the warmest days of the year. The lowest temperatures occur at night, with frequent records of 22°C at dawn. The three selected areas were considered strategic for regular phlebotomine captures due to the high incidence of human CL cases in the neighboring population. Follows a detailed description of each area:

Area I - Chico Mendes Municipal Park, situated in the Vila Acre district, with one collection point (10° 02’ 135” S, 67° 47’ 716” W). The park occupies an area of 52 ha, located beside the highway AC-040, 10 km from the city of Rio Branco. It is considered one of the last remaining areas of primary forest in the city, with very important representative species of fauna and flora.

Area II – Bosque district, with two collection points (09° 55’ 802” S, 67° 51’ 763” W and 9° 55’ 820” S, 67° 51’ 340” W). This represents an urban area located near the center of Rio Branco, where all houses were constructed very close to the Amazon forest, with domestic and occasional wild animals present in the peridomicile.

Area III – Moreno Maia settlement project situated beside the Transacreana Road, with two collection points (10° 10’ 357” S, 67° 55’ 505” W and 10° 10’ 514” S, 67° 55’ 706” W). It is characterized as a rural area far away from the center of Rio Branco, where the few existing residences are positioned near the forest. Domestic animals are kept in the peridomicile.

## Results and discussion

**Figure 2:**
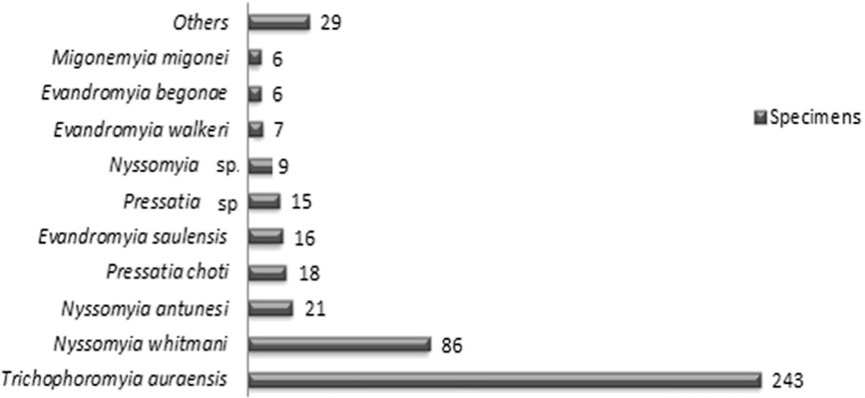
**Percentage distribution of sand fly specimens.** Total of sand fly specimens collected in Rio Branco municipality, state of Acre, from April 2011 to April 2012, using HP light traps distributed in four residential and one recreational areas.

During 13 months (April 2011 to April 2012) 456 sand flies were collected, 200 males and 256 females distributed in 23 species (Table 1). As demonstrated in Figure 2, *Trichophoromyia auraensis* was the most abundant species represented by 243 individuals, followed by *Nyssomyia whitmani* with 86 specimens. Although the number of sand flies trapped was relatively low, this region has a large variety of species, as observed in other areas of primary forest [8, 13].

**Table 1:** **Sand fly species collected in the Rio Branco municipality separated by type of environments**

	Area I (Urban)				Area II (Rural)		
Environment	Peridomicile			Peridomicile			Total
Ecotopes	Chicken coop	Forest	Total	Chicken coop	Forest	Total	
Species	Number (N)	N	N	Number (N)	N	N	
*Trichophoromyia auraensis*	31	155	186	55	2	57	**243**
*Nyssomyia whitmani*	6	80	86				**86**
*Nyssomyia antunesi*		21	21				**21**
*Pressatia choti*		18	18				**18**
*Evandromyia saulensis*		16	16				**16**
*Pressatia* sp.		15	15				**15**
*Nyssomyia* sp*.*		6	6	3		3	**9**
*Evandromyia walkeri*	2	5	7				**7**
*Evandromyia begonae*		6	6				**6**
*Migonemyia migonei*	2	4	6				**6**
*Pintomyia serrana*		4	4				**4**
*Psychodopygus paraensis*		4	4				**4**
*Sciopemyia sordelii*	1	2	3				**3**
*Migonemyia pusilla*		3	3				**3**
*Pintomyia nevesi*		3	3				**3**
*Brumptomyia avellari*	1	1	2				**2**
*Micropygomyia acanthopharynx*	1	1	2				**2**
*Pintomyia odax* 1		1				**1**	
*Lutzomyia sherlocki*	1		1				**1**
*Pressatia calcarata*	1		1				**1**
*Micropygomyia micropyga*		1	1				1
*Pressatia duncanae*		1	1				1
*Bichromomyia flaviscutellata*		1	1				1
*Evandromyia bourrouli*		1	1				1
*Evandromyia bacula*		1	1				1
**Total**	**47**	**349**	**396**	**58**	**2**	**60**	**456**

In a rural settlement (Moreno Maia), due to the extreme difficulty to get in this area, only one night´s capture was performed and 60 sand flies were collected from two genera: *Trichophoromyia aurensis* and *Nyssomyia* sp.

**Figure 3:**
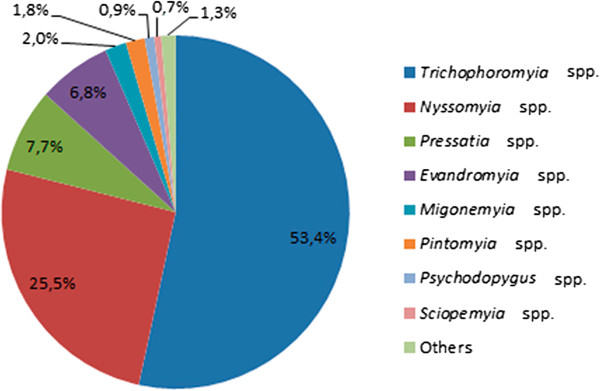
**Percentage distribution of sand fly genera.** Sand fly genera collected in Rio Branco municipality, state of Acre, from April 2011 to April 2012, using HP light traps distributed in four residential and one recreational areas.

In the urban areas, 396 sand flies representing 16 genera were captured, with *Trichophoromyia* and *Nyssomyia* the most prevalent genera, corresponding to 53% and 25% respectively of the specimens collected (Figure 3).

As observed in several Brazilian cities, construction of residential areas within forested regions in Rio Branco has had a negative impact on health through dissemination of infectious diseases. Uncontrolled construction has resulted in a series of environmental transformations, which have promoted the spread of cutaneous leishmaniasis in Rio Branco and others neighboring municipalities. Rapid changes in environmental conditions in many tropical regions, caused by habitat destruction, deforestation and associated urbanization processes, have enormous influence on insect vector populations and therefore, the transmission of the disease. While some species are subjected to extinction, others may become more abundant [4]. This aspect is not well understood in neotropical sand flies and better knowledge of the geographical distribution of sand fly species in the transmission of cutaneous leishmaniasis is still scarce for some regions, such as the state of Acre, where the disease is endemic [14–16].

There is strong evidence that the presence of domestic and wild animals nearby housing attracts a large number of sand flies, including species that act as leishmaniasis vectors, thus contributing to the increased risk of transmission in these areas [16, 17]. In the present study, a large number of chicken coops, dogs and a variety of species of birds were observed in the peridomicile of houses, beyond frequent inhabitant’s reports regarding the presence of wild animals, especially at night.

From an investigation carried out in 2008, spanning the municipalities of Bujari, Xapurí and Rio Branco in Acre state, the abundance of sand fly species found on the ground and in tree canopy was estimated. *Trichophoromyia auraensis*, *Ny. antunesi*, *Ny. whitmani* and *Ps. davisi* accounted for 66.95% of the specimens collected. *Nyssomyia whitmani* was the most abundant species*,* followed by *Ny. antunesi* and *Ps. davisi* [8]. Since then, no other study on sand fly fauna has been conducted in this region.

Among the species identified in our work, *Tr. auraensis, Ny. whitmani, Ny. antunesi, Pressatia choti, Evandromyia saulensis, Pressatia* sp*., Nyssomyia* sp*., Ev. walkeri, Ev. begonae, Mi. migonei, Pintomyia serrana, Ps. paraensis, Sciopemyia sordelii, Mi. pusilla, Pi. nevesi, Brumptomyia avellari, Micropygomyia acanthopharynx, Mi. micropyga, Pi. odax, Lutzomyia sherlocki, Pr. calcarata, Pr. duncanae, Bi. flaviscutellata, Ev. bourrouli,* and *Ev. bacula,* all of them have been previously reported in Rio Branco [8, 10].

The large number of *Trichophoromyia* spp. and *Nyssomyia* spp. collected in the present study corroborates the data from Azevedo *et al*. (2008) [8], concerning the abundance of these phlebotomine genera in Rio Branco and others municipalities from Acre state.

## Conclusion

In summary, we found that the sand fly fauna in the urban and periurban areas of Rio Branco is very diverse with 23 species, as observed in other areas of primary forest. Some species such as *Nyssomyia whitmani*, *Ny. antunesi* and *Bichromomyia flaviscutellata* are known vectors of parasites responsible for cutaneous leishmaniasis, and *Trichophoromyia auraensis* the putative vector in this Amazonian region.

## References

[1] Ministério da Saúde (2007). Manual de Vigilância da Leishmaniose Tegumentar.

[2] Rangel EF, Lainson R (2009). Proven and putative vectors of American cutaneous leishmaniasis in Brazil: aspects of their biology and vectorial competence. Mem Inst Oswaldo Cruz.

[3] Grimaldi G, Tesh RB, McMahon-Pratt D (1989). A review of the geographic distribution and epidemiology of leishmaniasis in the New World. Am J Trop Med Hyg.

[4] Lainson R, Shaw JJ, Silveira FT, Sousa AAA, Braga RR, Ishikawa EAY (1994). The dermal leishmaniases of Brazil, with special reference to the eco-epidemiology of the disease in Amazonia. Mem Inst Oswaldo Cruz.

[5] Sistema de informação de agravos de notificação/Secretaria de Vigilância e Saúde/Ministério da SaúdeTabulação de Dados - Acessado em 02.05.2013*)*. http://dtr2004.saude.gov.br/sinanweb/index.php

[6] Tojal AC, Cupolillo E, Volpini AC, Almeida R, Romero GAS (2006). Species diversity causing human cutaneous leishmaniasis in Rio Branco, state of Acre, Brazil. Trop Med Int Health.

[7] Tojal AC, Romero GAS, Cupolillo EA (2003). A diversidade das espécies causadoras de leishmaniose cutânea em Rio Branco - Acre. Rev Soc Bras Med Trop.

[8] Azevedo ACR, Costa SM, Pinto MCG, Souza JL, Cruz HC, Vidal J, Rangel EF (2008). Studies on the sandfly fauna (Diptera: Psychodidae: Phlebotominae) from transmission areas of American Cutaneous Leishmaniasis in state of Acre, Brazil. Mem Inst Oswaldo Cruz.

[9] Pugedo H, Barata RA, França-Silva JC, Silva JC, Dias ES (2005). HP: um modelo aprimorado de armadilha luminosa de sucção para a captura de pequenos insetos. Rev Soc Bras Med Trop.

[10] Galati EAB (2003). Morfologia e Taxonomia: Morfologia, terminologia de adultos e identificação dos táxons da América. Flebotomíneos do Brasil, Editora Fiocruz.

[11] Marcondes CB (2007). A proposal of generic and subgeneric abbreviations for Phlebotomine Sandflies (Diptera: Psycodidae: Phlebotominae) of the World. Entomol News.

[12] Instituto Brasileiro de Geografia (IBGE): http://www.ibge.gov.br/estadosat/perfil.php?sigla?=?ac: accessed in 11.03.2014

[13] Bejarano EE, Uribe S, Rojas W, Vélez ID (2002). Phlebotomine sandflies (Diptera: Psychodidae) associated with the appearance of urban Leishmaniasis in the city of Sincelejo, Colombia. Mem Inst Oswaldo Cruz.

[14] Martins AV, Silva JE (1964). Notas sobre os flebotomíneos do Estado do Acre, com a descrição de duas espécies novas (Diptera, Psychodidae). Rev Bras Biol.

[15] Arias JR, Freitas RA, Barrett TV (1984). A new sandfly in the subgenus *Nyssomyia (Diptera, Psychodidae)* from the Amazon Basin of Brazil. Mem Inst Oswaldo Cruz.

[16] Silva-Nunes M, Cavasini CE, Silva NS, Galati EAB (2008). Epidemiologia da Leishmaniose Tegumentar e descrição das populações de flebotomíneos no município de Acrelândia, Acre, Brasil. Rev Bras Epidem.

[17] Brazil RP, Morton IE, Ward RD (1991). Notes of the feeding habits of *Lutzomyia* (*Nyssomyia*) *whitmani* (Diptera: Psychodidade) in Ceará State, Northeast Brazil. Mem Inst Oswaldo Cruz.

